# Ultradurable Embedded Physically Unclonable Functions

**DOI:** 10.1021/acsami.4c01726

**Published:** 2024-03-21

**Authors:** Abidin Esidir, Sami Pekdemir, Mustafa Kalay, Mustafa Serdar Onses

**Affiliations:** † ERNAM - Nanotechnology Research and Application Center, Erciyes University, Kayseri 38039, Turkey; ‡ Department of Materials Science and Engineering, Erciyes University, Kayseri 38039, Turkey; § Graduate School of Natural and Applied Science, Materials Science and Engineering Program, Erciyes University, Kayseri 38039, Turkey; ∥ Department of Electricity and Energy, Kayseri University, Kayseri 38039, Turkey; ⊥ Department of Aeronautical Engineering, Faculty of Aeronautics and Astronautics, Erciyes University, Kayseri 38039, Turkey

**Keywords:** physically unclonable function, dry etching, durability, photoresist, electrospraying

## Abstract

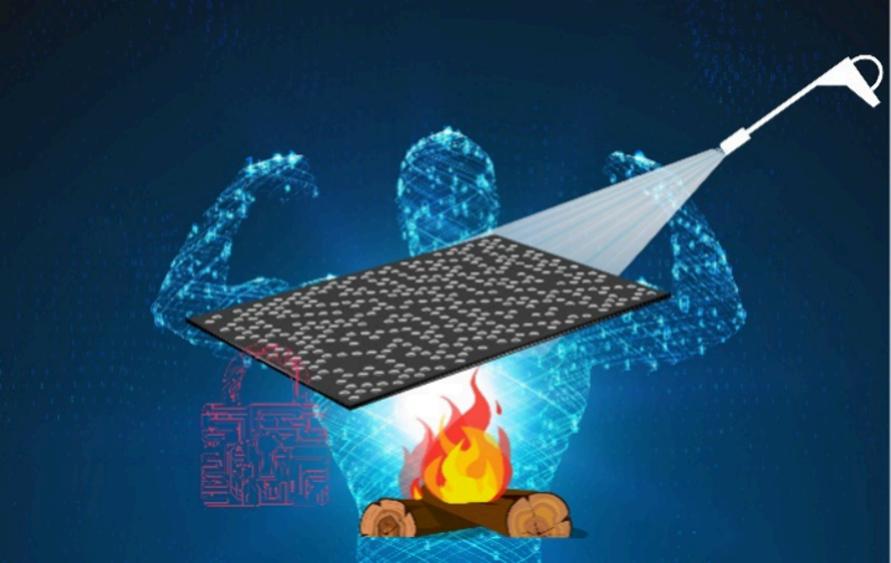

Physically unclonable functions (PUFs) have attracted growing interest for anticounterfeiting and authentication applications. The practical applications require durable PUFs made of robust materials. This study reports a practical strategy to generate extremely robust PUFs by embedding random features onto a substrate. The chaotic and low-cost electrohydrodynamic deposition process generates random polymeric features over a negative-tone photoresist film. These polymer features function as a conformal photomask, which protects the underlying photoresist from UV light, thereby enabling the generation of randomly positioned holes. Dry plasma etching of the substrate and removal of the photoresist result in the transfer of random features to the underlying silicon substrate. The matching of binary keys and features via different algorithms facilitates authentication of features. The embedded PUFs exhibit extreme levels of thermal (∼1000 °C) and mechanical stability that exceed the state of the art. The strength of this strategy emerges from the PUF generation directly on the substrate of interest, with stability that approaches the intrinsic properties of the underlying material. Benefiting from the materials and processes widely used in the semiconductor industry, this strategy shows strong promise for anticounterfeiting and device security applications.

## Introduction

Counterfeit products and identity breaches pose an increasing threat to the world economy, national security, and human health.^1^ With the advancement of technology, it is becoming increasingly difficult to provide security for personal data, critical documents, information, and objects. The highly digitized world has come with its own weaknesses, and cryptography practices that rely on mathematical functions and algorithms have become vulnerable to attacks of third parties. An attractive strategy is to benefit from physical systems in encoding.^2^ Physically unclonable functions (PUFs) make use of stochastically driven physical systems that produce a unique and unclonable response to an applied challenge.^3^ Each PUF is unique by analogy to the human fingerprint that consists of randomly oriented features. Earlier PUFs benefited from inherent variations in the response of electronic devices. Motivated by the accelerating needs of encoded surfaces in anticounterfeiting and authentication, a diverse range of processes and materials have been recently explored for generation of PUFs. Quantum dots,^4−6^ perovskite nanocrystals,^7^ luminescent materials,^8,9^ plasmonic nanoparticles,^10−12^ 2D materials,^13^ organic semiconductors,^14^ graphene,^15^ food-grade starch,^16^ self-wrinkling materials,^17,18^ self-assembly of polymers,^19^ light-emitting organic molecules,^20^ electronic fingerprints,^21^ laser-induced carbonization,^22^ and polymeric particles^23^ are good examples to recent reports. The rich menu of materials proposed for PUF applications provides viable options for the vastly diverse needs of these applications. In that regard, the durability of PUFs deserves special attention, particularly for applications where exposure to harsh conditions, such as extremely elevated temperatures, is possible. Previous efforts either did not even consider the durability or studied very modest conditions.

The typical PUF fabrication process involves deposition of materials on top of a solid material using wet chemical and vapor deposition methods.^24^ There are two limitations of this fabrication approach for the stability of PUFs. First, the stability of PUF is determined by the intrinsic material properties of the deposited materials. Quantum dots,^25^ and perovskite nanocrystals,^26,27^ for example, can lose their photoluminescence and plasmonic nanoparticles experience distortions in their shapes when heated at elevated temperatures. The second challenge associated with the PUF fabrication by deposition of materials is the ease of delamination of the active layer.^28,29^ The interfacial strength between the substrate and the active layer determines the stability of PUF. Even slight distortion of this interface can lead to issues in the accurate readout of the PUF response. New processing strategies are necessary to address these issues and enable the generation of PUFs that are resistant to extreme conditions.

In this study, we present extremely durable PUFs through the embedding of randomly positioned features. This strategy overcomes the limitations of PUFs fabricated by the deposition of materials. To demonstrate this strategy, we use SU-8, a negative tone photoresist. SU-8 cross-links when exposed to UV light and allows the generation of deep microstructures. To generate randomly positioned holes within SU-8, electrosprayed polymer features are utilized as photomasks. True physical randomness is achieved via the chaotic electrohydrodynamic instability-driven low-cost electrospraying process, in comparison to photolithography, which relies on digitally defined masks and clean rooms. Poly(2-vinlypyridine) (P2VP) features with varied dimensions are deposited with spatial randomness on a film of SU-8. Upon UV light exposure, electrosprayed polymeric features act as conformal photomasks and prevent cross-linking of the underlying SU-8 film. Washing in an appropriate organic solvent simultaneously removes the polymer and underlying uncross-linked SU-8 film and yields randomly positioned holes. To transfer these random features to the underlying silicon substrate, a reactive ion etching process was utilized. The time of etching determined the hole depth, which is a key parameter for the durability of the embedded PUFs. After the transfer process, the cross-linked photoresist was removed by burning in a muffle furnace. This fabrication process enabled mechanically and thermally durable embedded PUFs. Authentication was performed via binary keys derived from optical images and feature matching algorithms.

## Experimental Section

### Materials

Silicon wafers (⟨100⟩, N/Phos) were purchased from University Wafer. The SU-8 negative-tone photoresist was purchased from Kayaku Advanced Materials. P2VP (40000 g/mol) was purchased from Polysciences Inc. *N*,*N*-Dimethylformamide (DMF), chloroform, chlorobenzene, and toluene were purchased from Sigma-Aldrich. Acetone was purchased from Merck.

### Fabrication of Embedded PUFs

SU-8 was spin-coated onto the silicon substrate at 4000 rpm for 55 s. The films were heated on a hot plate at 45 °C for 3 min. Polymer features with random positions were obtained by electrospraying (Holmarc HO-NFES-040) P2VP homogeneously dissolved in a chlorobenzene/DMF (9/1 v/v) solvent mixture. A 30 wt % P2VP solution was electrosprayed for 1 min via a metal-tipped syringe at an electrostatic potential of 12–15 kV, a feed rate of 1 mL/h, and a collector–nozzle tip distance of 15 cm. SU-8 thin films with electrosprayed polymeric structures were exposed to a 400 W UV halogen lamp (365 nm wavelength) (Uniterm) for 10 min.^30^ Electrosprayed polymeric structures at randomized positions acted as photomasks and prevented UV exposure of the underlying SU-8 film, whereas the photoresist was cross-linked in unmasked regions. The development of the sample was performed by immersion in chlorobenzene for 1 min. During the development step, the electrosprayed polymer structures and the unexposed SU-8 were simultaneously removed from the substrate, yielding randomly positioned holes in the SU-8 thin film. To transfer the random patterns to the underlying substrate, inductively coupled plasma reactive-ion etching (ICP–RIE) was used. The etching was performed using a mixture of the following gases: 20 sccm SF_6_, 80 sccm CHF_3_, and 20 sccm O_2_ in an ICP-RIE tool (SI 500, SENTECH). The ICP source was water-cooled and featured a planar triple antenna with a power output of up to 1200 W. The plasma source was isolated from the chamber by using an alumina plate supported by a quartz plate. The bottom electrode consisted of stainless steel and had a power of 300 W. The etching operation was executed at a temperature of 20 °C, a pressure of 5 mTorr, an RF power of 15 W, and an ICP power of 200 W. To control the depth of embedded features, the etching time was varied from 150 to 570 s. ICP-RIE removed the underlying silicon, which is not protected by SU-8, thereby leading to an effective transfer of PUFs to the substrate. After etching, the remaining SU-8 was removed by burning at 500 °C in a muffle furnace.^31^

### Characterization

An upright research microscope (Axio Imager 2, ZEISS) was used to acquire optical images of the samples. The typical exposure time was 160 ms. The morphology of the features was imaged by using scanning electron microscopy (SEM, EVO LS10, ZEISS) operated at 25 kV. Features were characterized by using an energy dispersive X-ray spectrometer (EDX, Bruker). The infrared spectrum was obtained using a PerkinElmer 400 Fourier transform infrared spectrometer with a MIRacle attenuated total reflection accessory (Pike Technologies). Depth and height profiles of the features were obtained by atomic force microscopy (AFM, 3000 Flex, Nanosurf). The Alpha M+ Raman spectrometer, a confocal micro-Raman microscope from Witec, was used for chemical analysis. The laser power was set to 0.5 mW, and a high-precision 100× microscope objective with a numerical aperture of 0.90 was used. The instrument’s intensity mapping setup involved collecting data at 40 × 40 points within 20 × 20 μm^2^ areas using a 500 nm step, and each point was measured for 0.1 s. Raman mapping images were generated by filtering bands positioned at 1005 and 1608 cm^–1^.

### Stability

The thermal and mechanical stabilities of embedded PUFs were evaluated by three different tests. A continuous water impact test was performed at an impact pressure of 10.48 kPa for 45 min with embedded PUFs positioned 30 cm below the water source and tilted for 45°. In the sand impact test, embedded PUFs were subjected to sand particles with a mass of 200 g freely falling from a height of 20 cm. The thermal stability of the embedded PUFs was probed by heating them at 1000 °C for 1 h in a muffle furnace. The furnace was heated at an acceleration rate of 10 °C per min. Optical microscopy images were taken before and after the tests and analyzed as described in the following section.

### Processing of Images and PUF Analysis

A code written in MATLAB was used to generate binary keys from optical microscopy images measuring 2752 pixels by 2208 pixels. These images were transformed from the RGB color space to the LAB color space. Subsequently, the optical microscopy images underwent processing using inversion, debiasing, and noise reduction algorithms. The von Neumann debiasing algorithm was applied to the keys; 256 (16 × 16) bit-long PUF keys were obtained by converting the original images to binary codes. In this binary representation, black pixels (0-bit) denote the embedded PUFs on the surface. The Oriented FAST and Rotated BRIEF (ORB) feature matching algorithm was used in the analysis, authentication, and matching of images taken at different conditions. The recognition score was calculated based on successful matching of feature points.

## Results and Discussion

### Fabrication of Embedded PUFs

**Figure 1:**
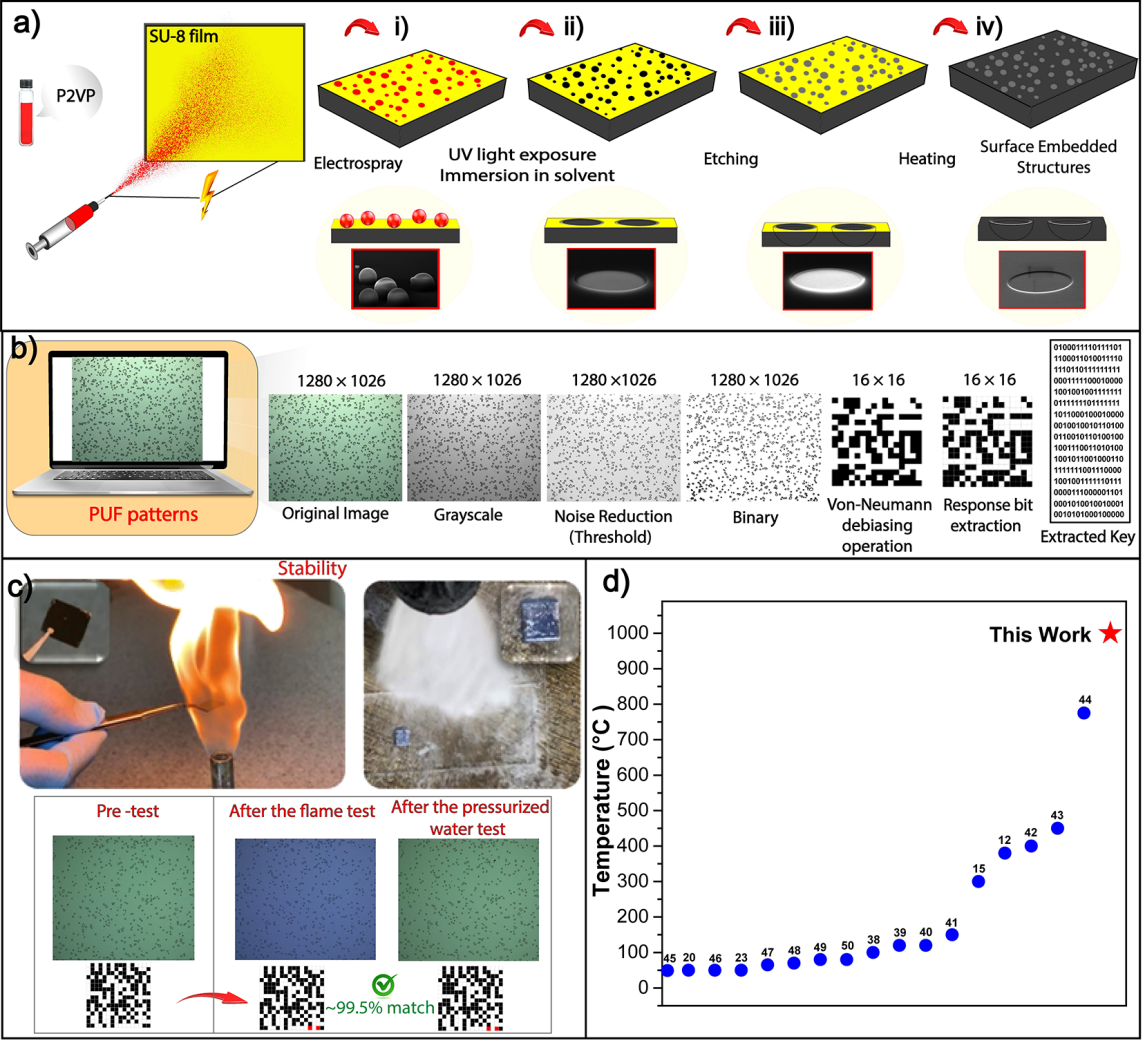
Ultradurable PUFs. (a) Schematic description of the fabrication process. (b) Binary key generation process. (c) Flame and pressurized water stability tests of ultradurable embedded PUFs. (d) Comparison of the thermal stability of PUFs with the literature.^12,15,20,23,38−50^

Figure 1a shows the step-by-step fabrication process and SEM images of embedded PUFs. A negative-tone photoresist, SU-8,^32^ was spin-coated onto the silicon substrate. Randomly positioned features with a broad size distribution were generated by electrospraying P2VP (Figure 1a,i). This randomness and distribution are perfectly suited for PUF applications and emerge from the chaotic electrohydrodynamic process.^33−35^ These polymer features acted as a photomask and prevented UV-light exposure to the underlying SU-8 film. The conformal contact of the polymer features with the SU-8 film without any gap in the out-of-plane direction was key to minimizing the diffraction effects. UV light exposure resulted in selective cross-linking of SU-8 at regions that are not protected by the electrosprayed polymer features.^36^ P2VP and uncross-linked SU-8 were removed by washing in chlorobenzene. As a result, holes were created at random positions in the SU-8 thin film (Figure 1a,ii). The randomly positioned holes were then transferred to the underlying silicon substrate via etching (Figure 1a,iii). ICP-RIE,^37^ also known as dry etching, was used for the pattern transfer. The depth of the holes was tuned by varying the etching time. Only the holes in the SU-8 thin film were transferred to the silicon substrate, whereas the areas of the substrate covered with the photoresist were not affected by the etching process (Figure 1a,iii). The cross-linked SU-8 film was then removed by burning^31^ in a muffle furnace at 500 °C for 1 h. This process resulted in PUFs embedded in the underlying silicon substrate. The optical microscopy image of the surface was then used as the response of PUF. These microscopy images were processed to generate the binary keys. Optical microscopy images (1280 × 1026 pixels) were converted from the RGB color space to the LAB color space (Figure 1b). These images were then converted to grayscale and processed by using inversion, dehazing, and noise-reduction algorithms. Finally, 256-bit long (16 × 16) binary keys were obtained. A remarkable feature of the embedded PUFs is their extremely high stability. In principle, this approach yields PUFs with stability that is defined by properties of the substrate material. Surface-embedded PUFs were subjected to flame and high-pressure water impact tests (Figure 1c and Supporting Video 1). The binary keys derived from optical microscopy images taken before and after the durability test were almost identical. Figure 1d presents a comparison of our work with recent PUFs in terms of the maximum reported temperature that PUF can withstand. Our strategy enables extreme stability even when the sample was heated at 1000 °C and exposed to flames.

**Figure 2:**
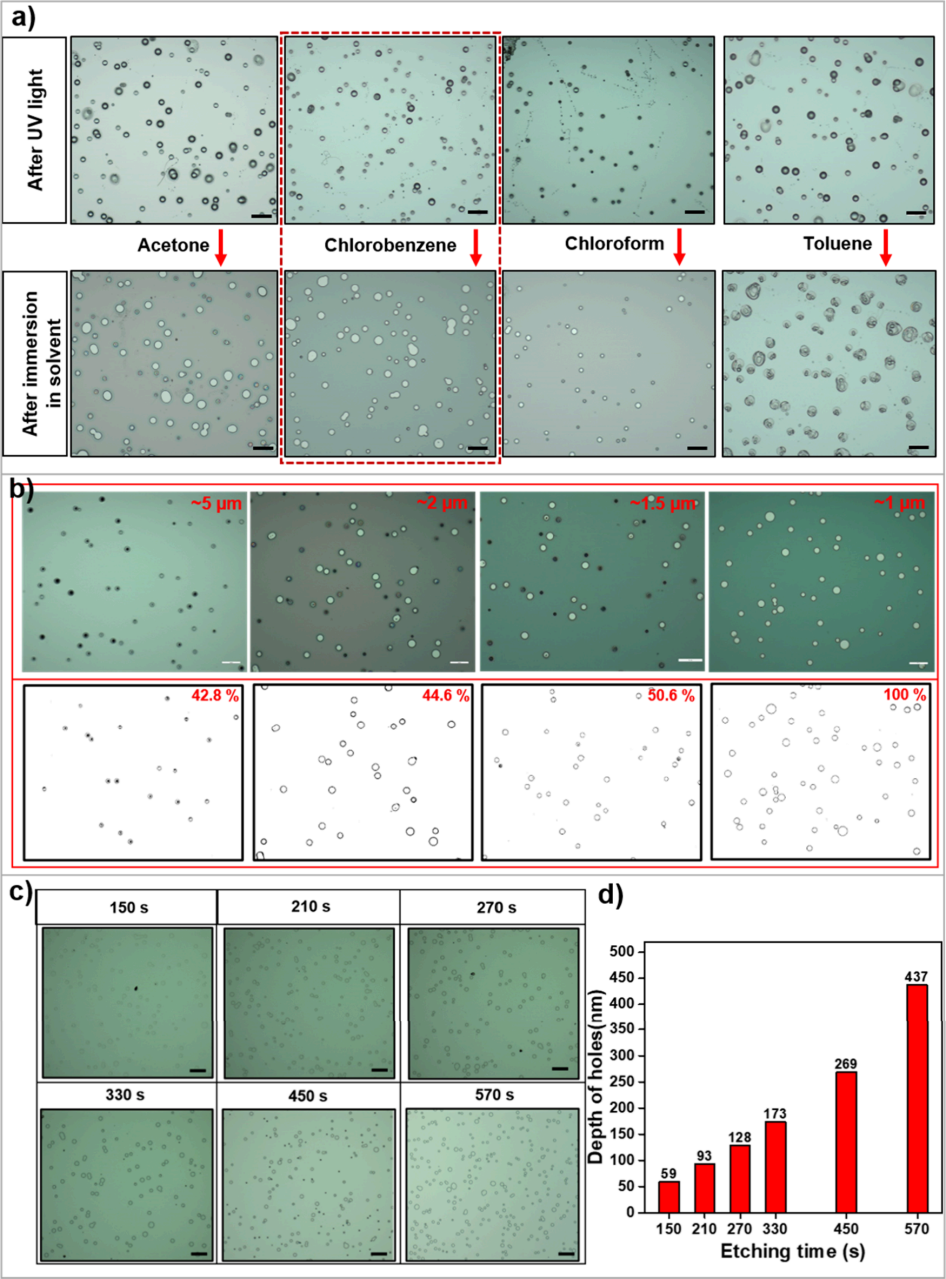
Effects of processing conditions. (a) Optical microscopy images of polymeric structures electrosprayed on SU-8 thin films after UV light exposure and washing in different solvents. Scale bars: 50 μm. (b) Optical microscopy images (top row) of the SU-8 films with varied thicknesses following the hole opening process consisting of electrospraying, UV light exposure, and washing in chlorobenzene. The thickness of SU-8 films is given at the top of images. The bottom row shows the positions of fully open holes and percentage of the hole opening. Scale bars: 50 μm. (c) Optical microscopy images of the substrate following ICP-RIE etching and removal of SU-8. Scale bars: 100 μm. The etching times are given at the top of each image. (d) Depth of holes derived from AFM imaging for each etching time.

The determination of the appropriate solvent for the removal of electrosprayed P2VP features and uncross-linked SU-8, the thickness of the SU-8 film, and etching time were important for the effective opening of holes. Figure 2a presents optical microscopy images of the SU-8 film following electrospraying, UV exposure, and washing steps. When washed in toluene, the holes did not form. In the case of washing with acetone and chloroform, the holes were partially opened. Chlorobenzene resulted in complete opening of the holes due to the effective removal of P2VP and uncross-linked SU-8. Subsequent studies were conducted using chlorobenzene as the solvent for the washing.

The second important parameter for the effective opening of holes is the thickness of the SU-8 film (Figure 2b). SU-8 films of varied thicknesses were produced by adjusting the rotational speed in the spin-coating process. At high thicknesses of the SU-8 film, the holes were not fully open. At a thickness of ∼5 μm, for example, less than 50% of the electrosprayed features formed holes in the film. The SU-8 film with a thickness of ∼1 μm prepared by spin-coating at 4000 rpm resulted in complete opening of the holes. Subsequent studies were conducted at a thickness of 1 μm. The etching time is a convenient means for controlling the depth of the holes. Figure 2c shows the depth of holes derived from AFM imaging for etching times of 150, 210, 270, 330, and 450 s. The hole depth scales with the etching time and determines the contrast in optical imaging and durability of the embedded PUFs as discussed later. Note that there is a slight variation in the density of the holes. This variation is due to localized variations in the deposition of polymer droplets with electrospraying. This type of variation contributes to the randomness and unclonability of the PUFs fabricated by electrospraying.

### Characterization of Embedded PUFs

**Figure 3:**
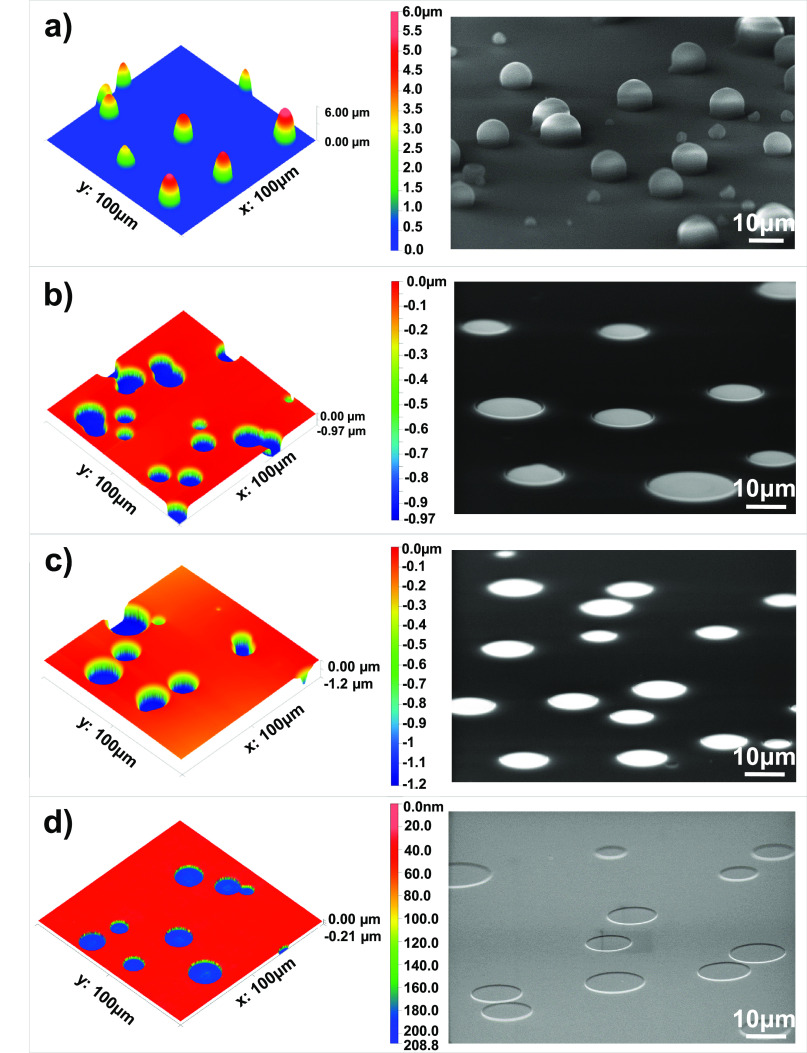
AFM and SEM images of the surface at each fabrication step. (a) After electrospraying of P2VP features over the SU-8 film. (b) Upon UV exposure and washing in chlorobenzene. (c) After ICP-RIE etching. (d) Embedded PUFs after removal of the remaining cross-linked SU-8 by burning in an oven.

Figure 3 presents a step-by-step evolution of the embedded PUFs probed by AFM and SEM imaging. The randomly positioned features fabricated by electrospraying exhibited a spherical morphology with limited spreading over the substrate (Figure 3a). The spherical morphology with limited contact with the solid interface is probably due to the relatively low surface energy of SU-8 emerging from the phenyl rings in the chemical structure.^51^ Upon UV light exposure and washing in chlorobenzene, holes appear at random locations defined by the electrosprayed P2VP features (Figure 3b).

The depth of holes was 0.97 μm, which was a good match with the thickness of the SU-8 film. The cross-linked SU-8 did not dissolve in the solvent and remained intact during washing (Figure 3b). The depth of holes increased with the ICP-RIE etching process to an extent that depends on the etching time (Figure 3c). The etching for 360 s, for example, resulted in a depth of ∼210 nm (Figure 3d). AFM measurements (Supporting Information, Figure S5) showed that the etching rates of silicon and SU-8 were 26 and 40 nm/min, respectively. Despite a slightly higher etching rate of SU-8 in comparison with silicon, a 1 μm-thick SU-8 is sufficient for effective opening of holes. There was a slight difference in the lateral dimensions of electrosprayed polymer features and holes formed after the washing, dry etching, and burning steps. Since only the embedded holes formed in the final step are used in the authentication process, the differences in the surface features after each step are irrelevant to the PUF metrics. These results demonstrate the feasibility of using electrosprayed P2VP structures as masks in the practical and tunable generation of PUFs without the need for complex infrastructure.

**Figure 4:**
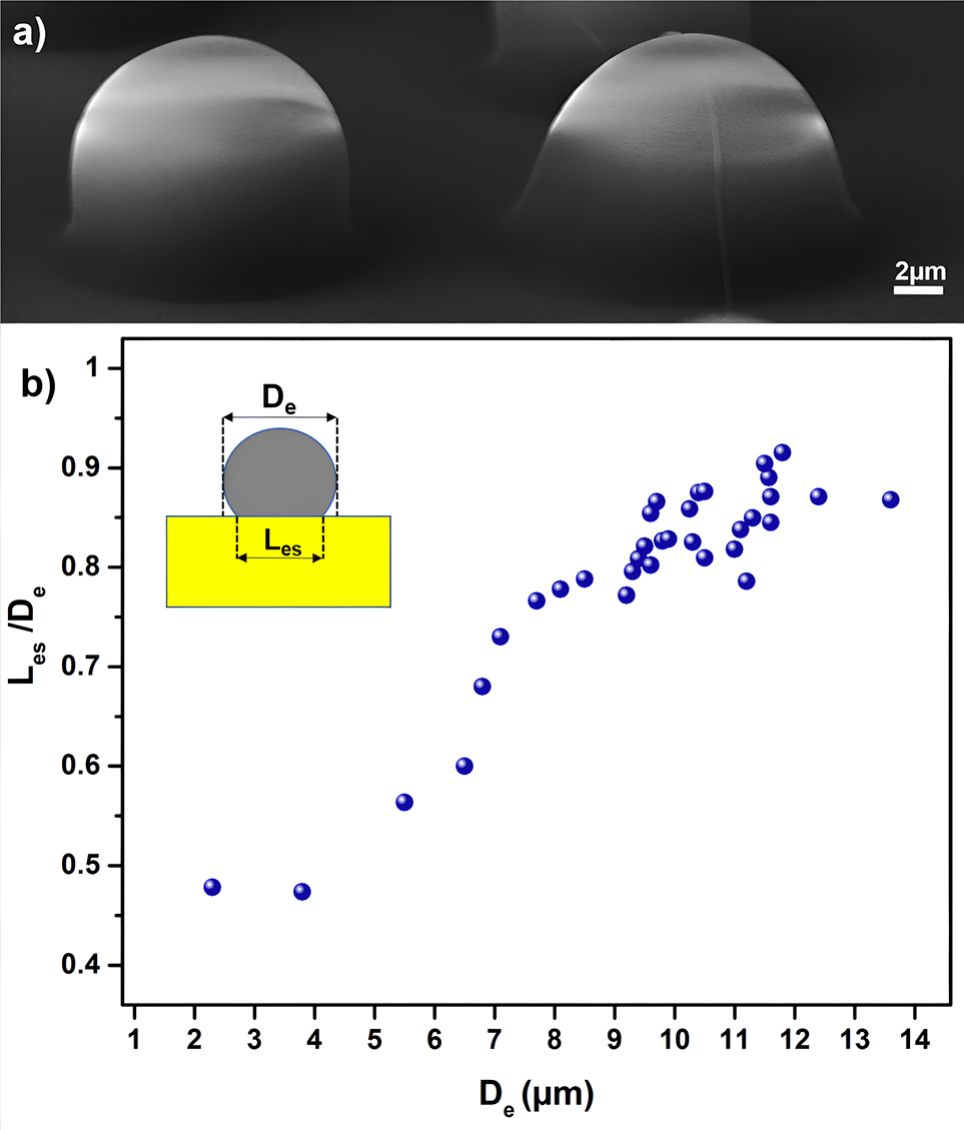
Interaction between electrosprayed polymer features and the substrate. (a) Cross-sectional SEM images of the electrosprayed polymer on top of the SU-8 film. (b) Relationship between the diameter of the feature and the length of the interface.

The interaction between electrosprayed polymer features and the SU-8 film is of immense importance in preventing UV light exposure and, thus, opening the holes. *L*_es_, the length of the interface formed between electrosprayed polymer features and SU-8, plays a critical role in the determination of the hole diameter. Cross-sectional SEM images (Figure 4a) revealed that *L*_es_ depended on the diameter (*D*_e_) of the electrosprayed polymer feature. Systematic examination of the cross-sectional SEM images of electrosprayed polymer features of varying diameters showed that the *L*_es_/*D*_e_ ratio increased as a function of *D*_e_ (Figure 4b). The *L*_es_/*D*_e_ ratio becomes much less than 0.6 for electrosprayed features with diameters of less than ∼7 μm. On the other hand, the *L*_es_/*D*_e_ ratio exceeded 0.9 for large electrosprayed polymer features due to spreading. The size-dependent variation of this ratio likely emerges from the interfacial forces and the amount of solvent trapped within the features. The high solvent content in the large features causes spreading over the substrate.^52,53^

**Figure 5:**
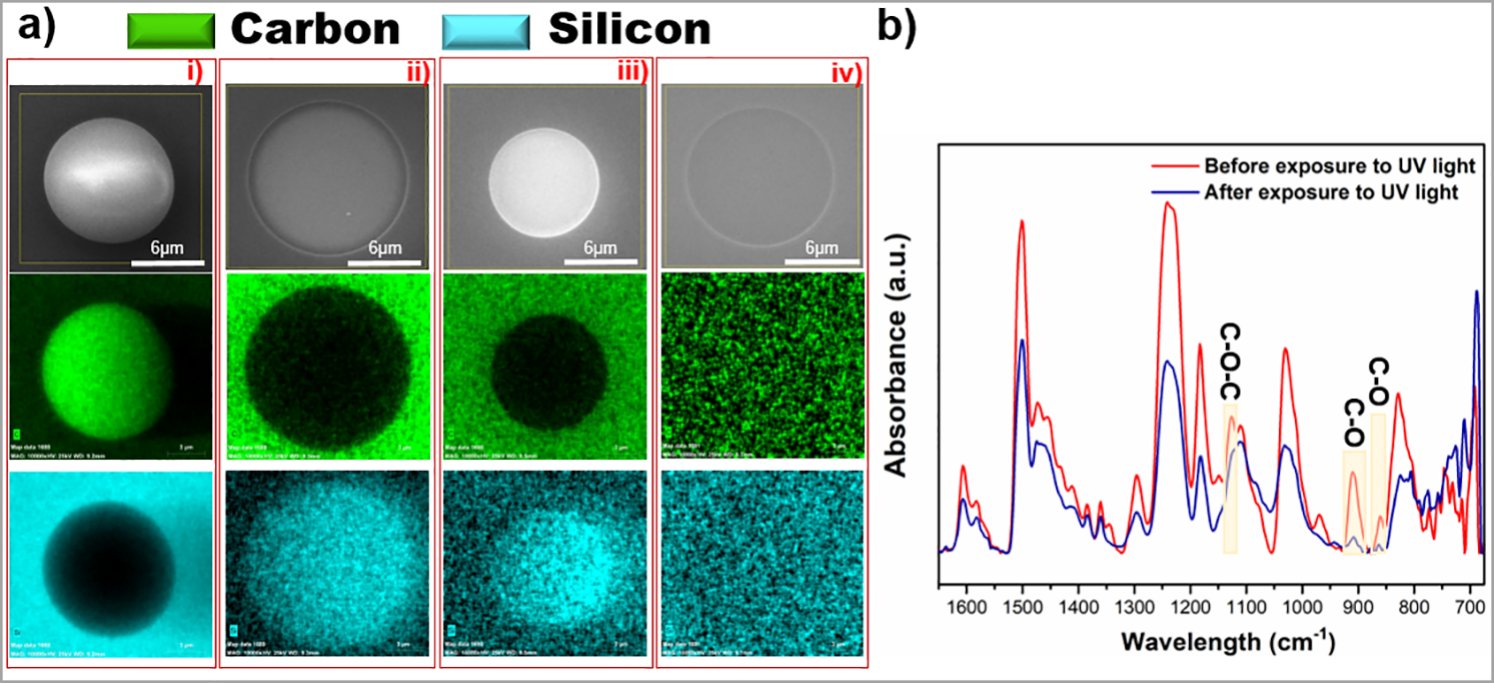
Chemical and structural characterization. (a) SEM and EDX mapping images after (i) electrospraying the polymeric structures on the SU-8 thin film, (ii) development in chlorobenzene, (iii) ICP-RIE etching, and (iv) removal of the SU-8 thin film by burning. (b) FT-IR spectra of SU-8 thin films before and after UV light exposure.

Chemical and structural characteristics are presented in Figure 5. SEM images and EDX mapping of carbon and silicon elements clearly support the evolution of embedded PUFs. After electrospraying, the feature is mostly composed of a carbon element as a result of the microscopic P2VP droplets placed over the SU-8 film (Figure 5a,i). Note that EDX mapping images are based on relative composition of the surface, i.e., the brighter regions have a higher concentration of that element, whereas the dark regions do not necessarily confirm the absence of the element. Raman mapping imaging (Figure S1) performed via scanning along the out-of-plane direction further identifies the presence of P2VP features over the SU-8 film. Following the washing step, the chemical contrast in the EDX mapping image has exhibited a reversal as a result of the removal of the carbon-containing P2VP and SU-8 from the feature (Figure 5a,ii). ICP-RIE etching further sharpened the contrast in the mapping image. The increased height contrast between carbon-containing SU-8 and etched silicon resulted in brighter spots in the SEM image (Figure 5a,iii). Finally, following burning, SEM and EDX mapping images show that the SU-8 thin film can be removed without damaging the silicon substrate (Figure 5a,iv). Chemical characterization of the surface-embedded PUFs was performed using Fourier transform infrared spectroscopy (FT-IR). The corresponding peaks in the FT-IR spectrum (Figure 5b) show the effect of cross-linking. The peaks at 861 and 910 cm^–1^ were attributed to the C–O stretching of the epoxy groups.^54,55^ The peak at 1128 cm^–1^ is the absorption peak, indicating the presence of an ether bond. This is attributed to the C–O–C stretching in the ether bond.

### PUF Metrics

**Figure 6:**
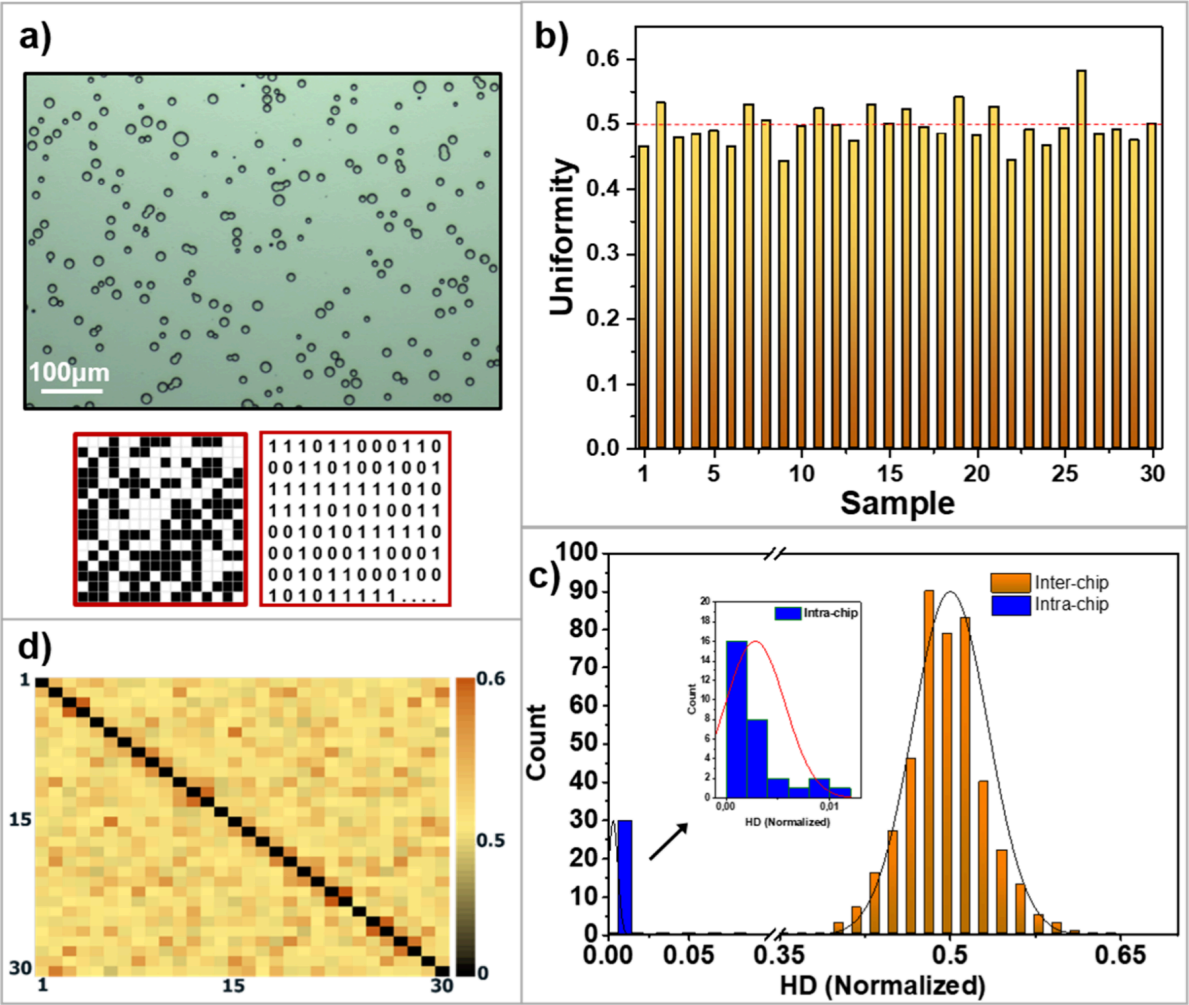
Key generation and extraction of PUF parameters. (a) Representative optical microscopy image of embedded PUFs. Binarization and size reduction of images for the generation of a security key. (b) Uniformity of bits obtained from 30 different PUF keys. (c) HD_INTRA_ and HD_INTER_ distributions. The distributions of HD_INTRA_ and HD_INTER_ are presented on the left and right, respectively. (d) Pairwise comparison map of interchip HD values of 30 PUFs.

To assess the performance of embedded PUFs, optical microscopy images were converted to binary keys. Figure 6a shows a representative optical microscopy image and the resulting 256-bit long keys. In this binary representation, black pixels (0-bit) correspond to the embedded PUFs on the surface, while white pixels (1-bit) represent the background areas. To achieve an equal distribution of 0-bits and 1-bits, two measures were taken. First, the electrospraying conditions^23^ were properly selected to generate polymer structures with a uniform coverage of the substrate. In particular, the duration of electrospraying plays a critical role in surface coverage. Second, we used a debiasing algorithm to further improve the uniformity. Since raw responses from physical systems can be uneven due to various physical characteristics, it is common for binary keys to exhibit an imbalanced proportion of 0-bits and 1-bits. von Neumann debiasing is commonly used to balance the uneven responses of the physical systems. This debiasing process was used to generate binary keys for 30 different PUFs (Supporting Information, Figure S2). The key PUF metrics include uniqueness, reliability, uniformity, and randomness.^56^ The first parameter evaluated is uniformity (eq S1), which measures the even distribution of 1-bits and 0-bits within the keys. The average uniformity of the keys was 0.495, which is very close to the ideal value of 0.5 (Figure 6b). Uniqueness (eq S2) measures the ability to distinguish one PUF from another and is calculated based on the interchip Hamming distance (HD_INTER_). Reliability (eqs S3 and S4), on the other hand, measures the repeatability of PUF responses under varying conditions and is probed by the intrachip Hamming distance (HD_INTRA_). HD_INTER_ and HD_INTRA_ values are displayed in Figure 6c. HD_INTRA_ was computed from images obtained from 30 distinct PUFs under five different lighting conditions for each PUF (Supporting Information, Figure S3). The average HD_INTRA_, derived from 30 different PUF keys, follows a Gaussian distribution with a standard deviation (σ) of 0.002849 and a mean (μ) of 0.002806, closely approaching the ideal value of 0. The HD_INTER_ values are near the ideal value of 0.5 with a μ of 0.495 and a σ of 0.033. In Figure 6d, the intra- and interchip distributions do not overlap, indicating extremely low false positive and negative rates. For a cutoff threshold of 0.1651, the false positive and false negative rates were found to be 8.38 × 10^–16^ and 4.77 × 10^–14^, respectively.^57,58^ Additionally, a pairwise comparison map of the HD_INTER_ values between chips is presented in Figure 6d. The encoding capacity (eq S5) is calculated as 2^227^ by only considering the independent bits through degrees of freedom analysis (see details in the Supporting Information). Considering uniqueness, reliability, uniformity, and encoding capacity, the embedded PUFs exhibit satisfactory performance.

Randomness of the embedded features and resulting binary keys is perhaps the most important for the unclonable nature of the reported approach. The randomness of binary-bit sequences was confirmed by tests proposed by the National Institute of Standards and Technology (NIST).^59^ To assess randomness, seven different tests were conducted using 7680 bits of digitized keys obtained from 30 different PUFs. Table 1 lists the results of the randomness tests, which involve applying a chi-square (χ^2^) distribution test to compare the goodness of fit. To ensure statistical significance in the assessment of the *p*-value uniformity, at least 55 sequences were processed. In this study, 60 sequences, each consisting of 128 bits, were subjected to NIST tests. *p*-Values ≥ 0.01 were considered as random. All these tests confirmed the randomness of binary sequences generated from surface-embedded PUFs (Supporting Information, Table S1).

**Table 1:** Summary of the Randomness Tests of Binary Sequences Generated from Surface-Embedded PUFs

**NIST statistical test**	***N***	*M* or *m*	***p*-value**	**proportion**	**result**
frequency	128		0.46183	60/60	pass
block frequency	128	20	0.60579	57/60	pass
cumulative sums	128		0.52662, 0.53041	60/60, 60/60	pass
runs	128		0.31486	56/60	pass
longest run of ones	128	8	0.21520	60/60	pass
approximate entropy	128	2	0.51310	59/60	pass
serial	128	4	0.77070, 0.32932	60/60, 56/60	pass

### Stability of Embedded PUFs

**Figure 7:**
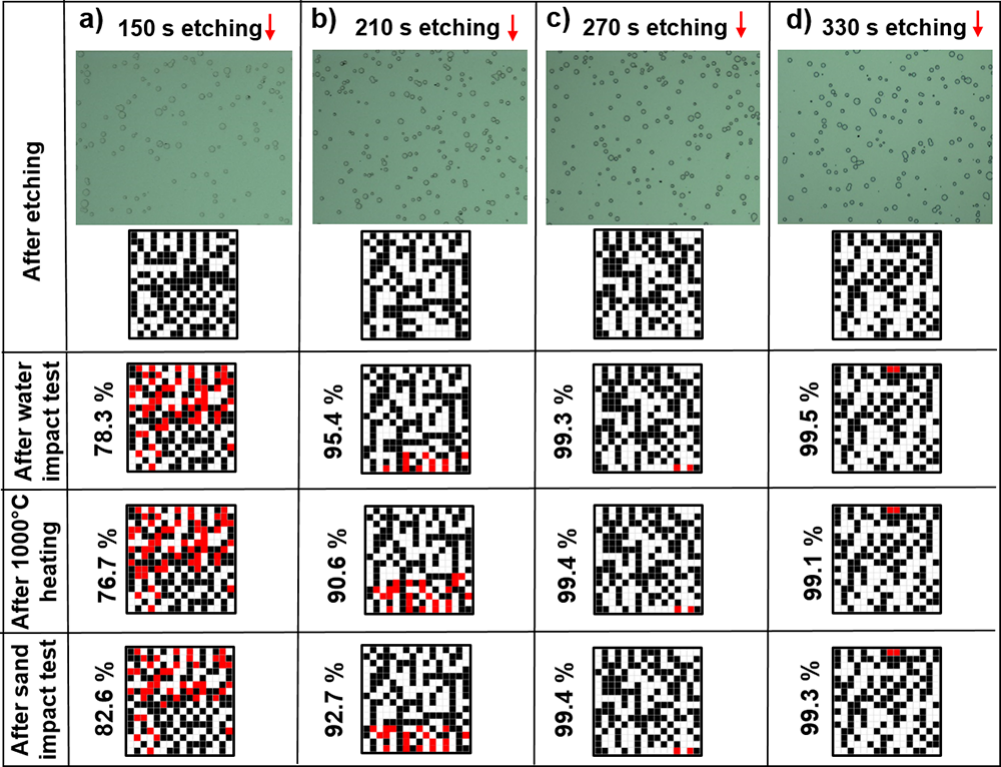
Stability of surface-embedded PUFs. Key generation was produced from optical microscopy images of surface-embedded microstructures prepared by etching for (a) 150, (b) 210, (c) 270, and (d) 330 s. After the stability tests, security keys were regenerated, and their similarity with the binary key generated before the test was compared. Water impact, sand impact, and high temperature durability tests were studied.

The thermal and mechanical stability of embedded PUFs were investigated using three different tests. Embedded PUFs of varied depth were fabricated by using etching times of 150, 210, 270, and 330 s. Optical microscopy images from the identical regions were taken before (Figure 7, top row) and after (Supporting Information, Figure S4) each stability test using a physical marker. Binary keys were generated from these optical microscopy images, and the percentage of the identical bits was calculated. To determine the mechanical durability, a continuous water impact test was performed. The samples placed at a 45° angle and 30 cm away from the water source were subjected to a continuous flow with a velocity of 4.6 m/s and an impact pressure of 10.5 kPa for 45 min (eqs S6 and S7 and Table S2). An additional test to probe the mechanical stability involves sand impact test, which is performed by dropping of sand particles from a height of 20 cm on the embedded PUFs. The thermal stability was studied by heating to an extremely elevated temperature of 1000 °C for 1 h. The depth of holes determined by the extent of etching plays a vital role in the stability of embedded PUFs for all three tests. At a short etching time of 150 s, there is a certain degree of distortion in the features, resulting in ∼20% alteration of the binary keys. The retention of the binary key sequences improves and becomes larger than 90% for an etching time of 210 s. Further etching of the sample substantially improves the stability of embedded PUFs with almost perfect (>99%) retention of the binary key sequences. Several factors contribute to these observations. First, with the increased depth of features, the image contrast becomes higher, ensuring the increased accuracy of matching between binary keys. Second, deeply embedding the features protects them against external effects. This kind of structural protection, for example, has been reported for superhydrophobic coatings.^60,61^ Embedded PUFs have successfully proven their reliability and longevity with excellent thermal and mechanical stability. Figure 1d shows the superior thermal stability in comparison to recent literature studies. Most of the previous PUF studies either did not consider mechanical stability or performed very modest experiments. The relatively harsh mechanical and thermal tests proposed in this study may guide future studies.

### Authentication via a Feature Matching Algorithm

**Figure 8:**
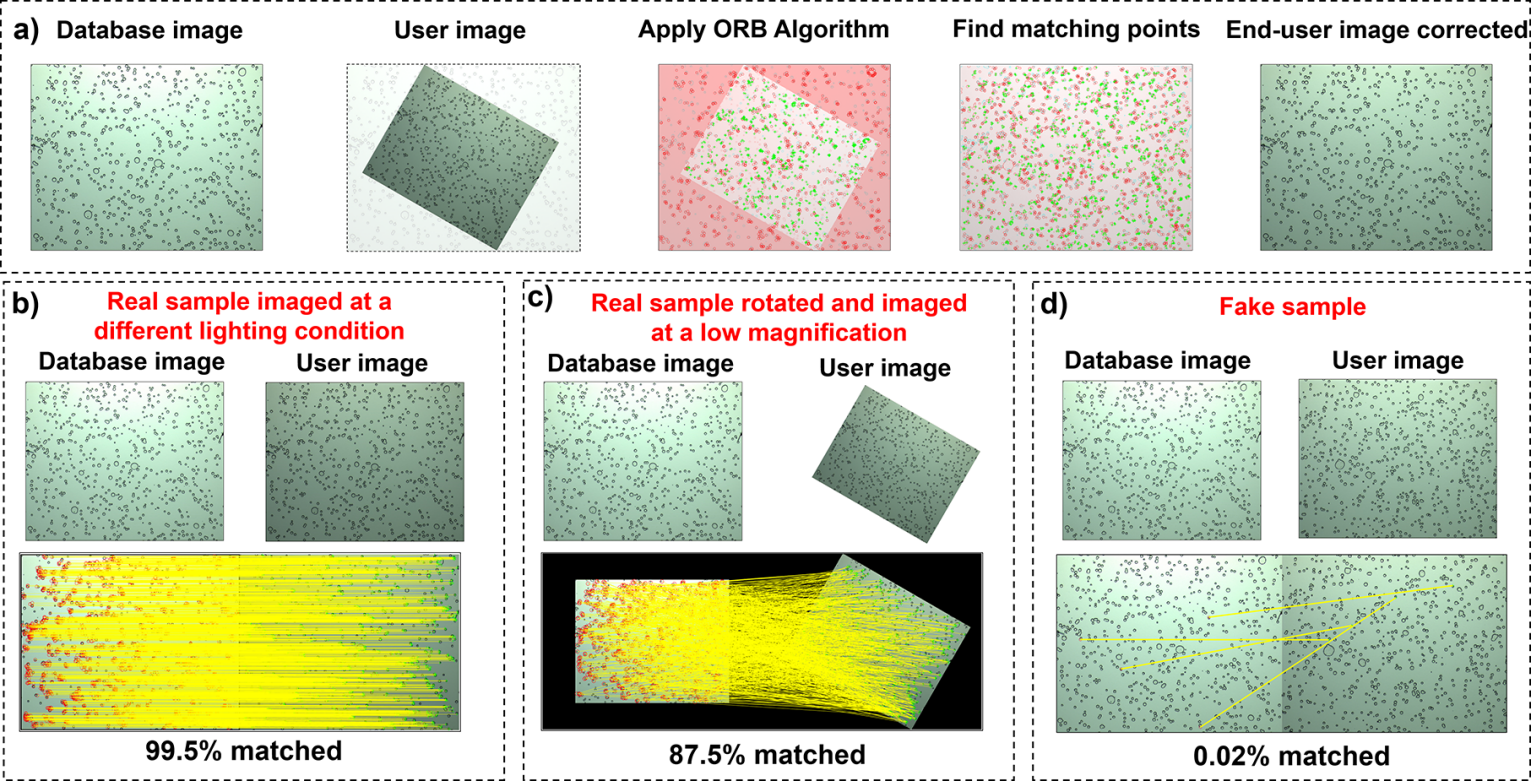
Authentication via a feature matching algorithm. (a) Proposed authentication approach using the ORB feature matching algorithm. (b–d) Feature matching for real and fake images for different imaging conditions. Yellow lines represent the matching of features between the database and the user image. Real sample imaged at different lighting conditions (b), real sample rotated and imaged at a low magnification (c), and fake sample (d).

The authentication of embedded-PUFs in real-world conditions requires further consideration of variations in the imaging conditions.^62^ The use of a feature matching algorithm, the ORB, is proposed for effective authentication of embedded PUFs. The ORB algorithm^63−66^ enables highly accurate and fast authentication of features. The ORB algorithm is a combination of the FAST key point detector and the BRIEF identifier, with some modifications. An additional advantage of ORB is its high recognition speed and low computational cost.^64,65^Figure 8a summarizes the proposed authentication process for an image taken by the user. The ORB algorithm is used to account for variations in the imaging conditions such as lighting, rotation, and magnification.^67^Figure 8b shows matching of features for three different cases. For the image stored in the database, 12,576 key points were detected. In the case of an image taken at a different lighting condition, 12,474 key points were successfully matched, resulting in a similarity rate of 99.5% (Figure 8b). Authentication was further challenged by using an image rotated by 30° and downsized by 30%. The ORB algorithm was again successful in authenticating 10,965 features with a similarity rate of 87.5% (Figure 8c). The matching key points dramatically reduced to only 4 for a fake image, yielding a similarity rate of 0.02% (Figure 8d). The significant difference in similarity values between genuine and fake labels allows for the determination of a suitable threshold value, ensuring authenticity even in images captured under diverse conditions. This threshold value is usually set depending on factors such as the application’s security level and error acceptability. In this study, the threshold value for ORB-based authentication was determined as 70%. The ORB algorithm is renowned for its rapid recognition and low computational demands, and the computational processing time for the ORB algorithm was less than 170 ms using a basic laptop computer. These results establish the effective authentication of embedded PUFs with the ORB-based feature matching algorithm. The authentication process is rapid, considering the 160 ms of exposure time used to acquire optical microscopy images. The most time-consuming step is the placement and registration of samples under the image acquisition system. Automated sample holders will be instrumental for practical implementation of such encoding systems.

The approach presented here is based on scalable processes that are commonly used in industrial environments. The semiconductor industry routinely uses processes such as light exposure, spin coating, and dry etching for large-scale chip fabrication. Electrospraying is a low-cost process based on bulk polymers and can be adapted for the continuous deposition of materials. These aspects show the promise of the presented strategy for high volume manufacturing. An additional point to consider is the difficulty in replicating these features. The access and expertise required for the dry etching process will challenge the exploitation of this strategy by counterfeiters. Although molding curable materials, such as cross-linkable PDMS, over such topographic features can generate replicas of surfaces, these methods will fail to create these features embedded over the target object and are therefore not able to achieve the extreme levels of stability. Future studies may further explore novel unlocking methods that prevent the unauthorized tempering of surfaces. Recently demonstrated advanced multilevel encoding schemes based on block copolymer films^47^ can be used to generate nanoscopic features within the holes to further challenge the duplication.

## Conclusions

Randomly positioned holes embedded in a substrate were demonstrated as a practical strategy to achieve highly durable PUFs. The stability of the embedded PUFs approaches the intrinsic material properties of the substrate when the holes are sufficiently deep. On silicon substrates, the embedded PUFs remain stable after heating at a temperature as high as 1000 °C and extended exposure to water and sand impact. The high level of stability is a consequence of fabricating PUFs that are free from additional materials deposited on the substrate. The proposed method ensures randomness via low-cost electrospraying-based deposition on a negative-tone photoresist. The process can be adapted to chip security applications as SU-8 and RIE processes are commonly employed for microfabrication.^68^ The stability of the embedded materials can be further improved by using high temperature-resistant ceramic materials.

## Supporting Information Available

Raman mapping and spectra, additional optical microscopy and AFM images, *p*-values for 30 keys, calculation of encoding capacity, equations used for the PUF analysis, and calculation of the pressure for the water impact test (PDF)
Video of stability tests (MP4)
