# Supplementary material for: Ultradurable Embedded Physically Unclonable Functions
Source: ACS Appl Mater Interfaces. 2024 Mar 21;16(13):16532–43. doi: 10.1021/acsami.4c01726 (PMC10995905; doi:10.1021/acsami.4c01726)
Supplement: Supplementary file 1 — am4c01726_si_001.pdf [file am4c01726_si_001.pdf]

Supporting Information

Ultradurable Embedded Physically Unclonable Functions

Abidin Esidir [1,2,3], Sami Pekdemir [1,5], Mustafa Kalay [1,4], Mustafa Serdar Onses [1,2,]*

[1] ERNAM - Nanotechnology Research and Application Center, Erciyes University, Kayseri, 38039, Turkey.

[2] Department of Materials Science and Engineering, Erciyes University, Kayseri, 38039, Turkey.

[3] Erciyes University, Graduate School of Natural and Applied Science, Materials Science and Engineering Program, Kayseri, 38039, Turkey.

[4] Department of Electricity and Energy, Kayseri University, Kayseri, 38039, Turkey.

[5] Department of Aeronautical Engineering, Faculty of Aeronautics and Astronautics, Erciyes University, Kayseri 38039, Turkey.

* Address correspondence to: onses@erciyes.edu.tr

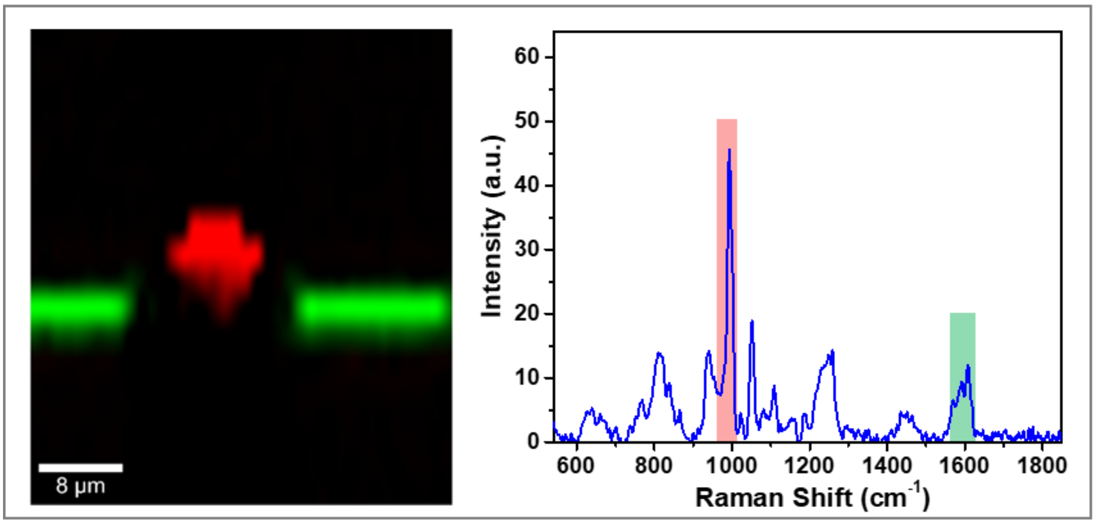

Figure S1. Raman mapping image (left) and Raman spectra (right) of electrosprayed P2VP features on a film of SU-8. The Raman mapping was performed with a through scan along the out of plane direction starting 10 µm above the surface and going down to a depth of 10 µm. To highlight the P2VP and SU-8 regions, the Raman mapping data was filtered at 1005 cm^{-1} and 1608 cm^{-1}, respectively.

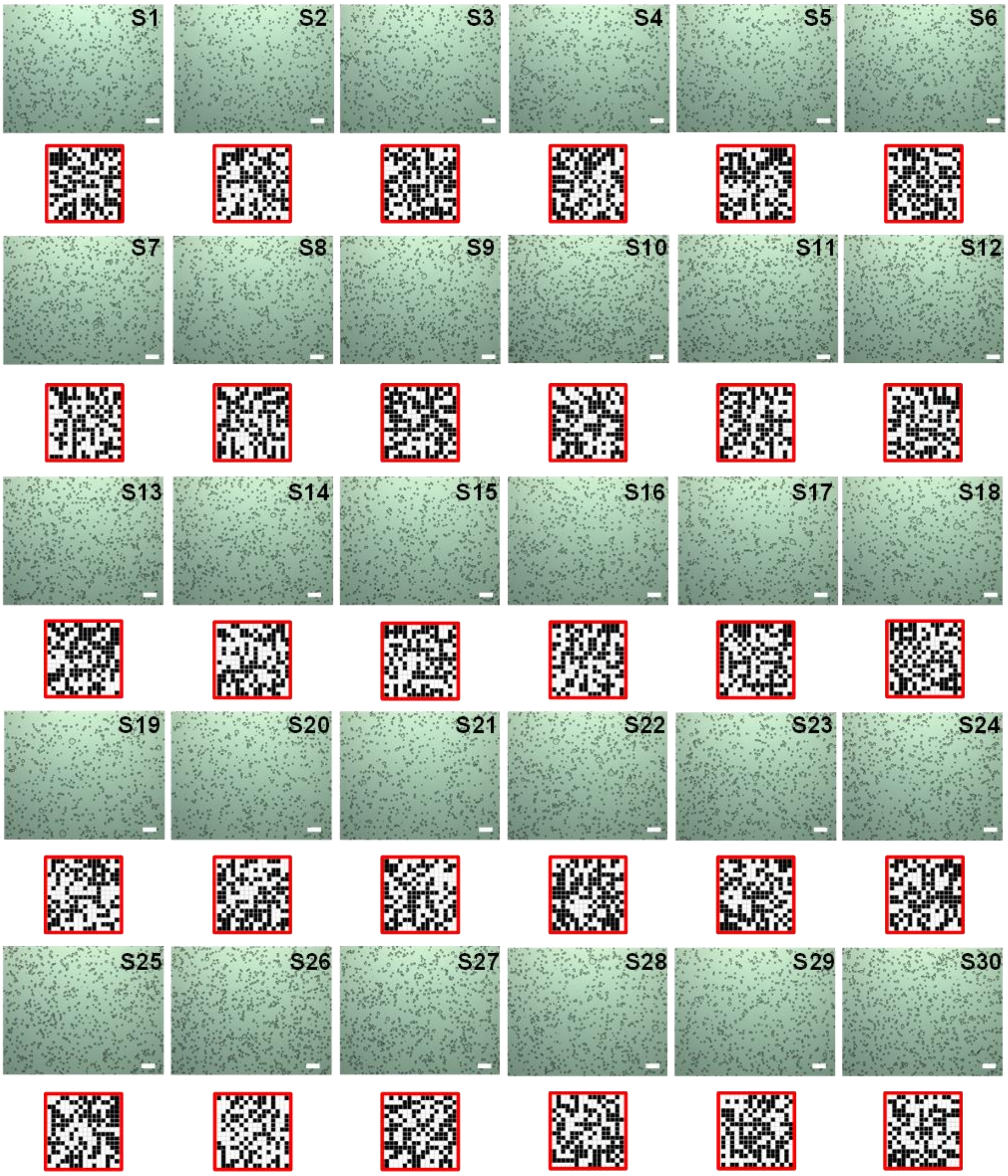

Figure S2. Optical microscope images of 30 different surface embedded PUFs and their corresponding binary keys. Scale bar: 100 μm.

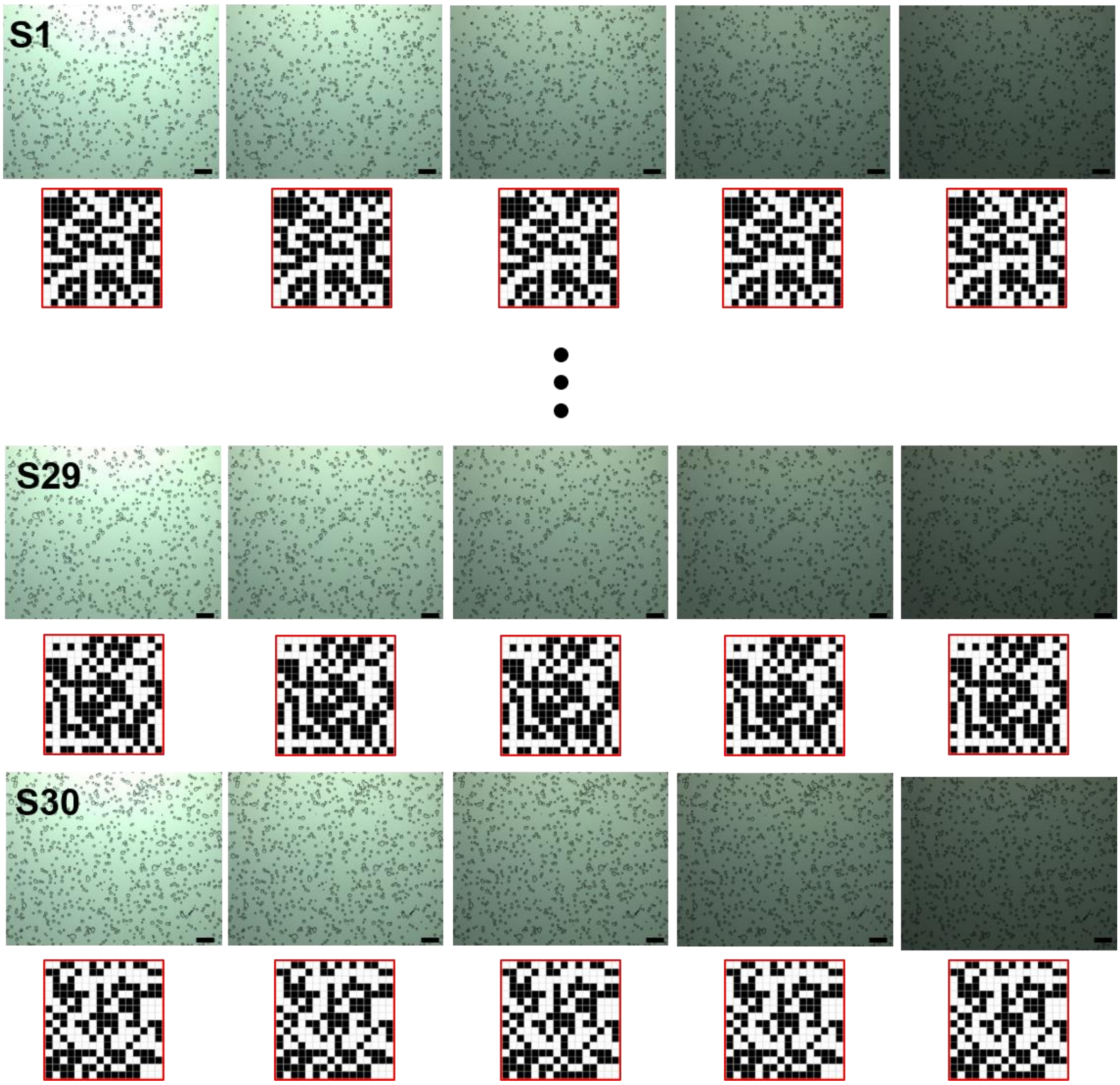

Figure S3. Representative images acquired from a selected region for varied illumination conditions. Exposure times are in the range of 70 to 190 ms. The keys extracted from these images were used in the calculation of HD_{INTRA}. Scale bars: 100 µm.

Table S1. *p*-values for 30 keys.

Sample	*P*-value	Sample	*P*-value
**S1**	0.258721	**S16**	0.490920
**S2**	0.283257	**S17**	0.852684
**S3**	0.490920	**S18**	0.618443
**S4**	0.661128	**S19**	0.190215
**S5**	0.755123	**S20**	0.614295
**S6**	0.256839	**S21**	0.383426
**S7**	0.349441	**S22**	0.089607
**S8**	0.849812	**S23**	0.804081
**S9**	0.062812	**S24**	0.285168
**S10**	0.899741	**S25**	0.852123
**S11**	0.456750	**S26**	0.009656
**S12**	0.950262	**S27**	0.614295
**S13**	0.421004	**S28**	0.756947
**S14**	0.354997	**S29**	0.487485
**S15**	0.901288	**S30**	0.951017

Uniformity

The uniformity for each key was calculated with the equation (S1).

$$\text{Uniformity} = \frac{1}{n}\sum_{i=1}^{n} r_{i,l} \times 100\% \quad \text{(S1)}$$

n: the number of bits

r_l: lth binary bit (0 or 1) of an n-bit response from a key

Uniqueness

Uniqueness was calculated using 30 keys obtained from the images presented in Figure S2 using equation (S2).

$$\text{Uniqueness} = \frac{2}{s(s-1)}\sum_{i=1}^{s-1}\sum_{j=i+1}^{s}\frac{HD(R_i,R_j)}{n} \quad \text{(S2)}$$

S: the number of keys from different chips

HD (R_i, R_j): Hamming distance between chips i and j

Normalized Hamming distance = Hamming distance divided by the number of bits, $\frac{HD(R_i,R_j)}{n}$

Reliability

Reliability was calculated using equations (S3) and (S4):

$$HD_{INTRA} = \frac{1}{m}\sum_{t=1}^{m}\frac{HD(R_i, R'_{i,t})}{n} \quad (S3)$$

m: number of samples studied under different conditions.

$$\text{Reliability} = 1.00\text{-}HD_{INTRA} \quad (S4)$$

Classic von Neumann Debiasing

The following procedure was used for debiasing:

- Consider the key is composed of consecutive pairs of bits.
- Discard the pair of bits in the case of bits consisting of 11 or 00.
- Retain the first bit, in the case of bits consisting of 10 or 01.

Encoding capacity

The encoding capacity, denoted as R^n, where R is the number of responses and n is the number of pixels. Herein R is 2 for binary PUFs. n is the number of pixels in an image. The accurate estimation of the encoding capacity requires only consideration of independent bits. The independent bits were calculated using the degrees of freedom (DoF) given by equation (S5):

$$DoF = \frac{\mu(1-\mu)}{\sigma^2} \quad (S5)$$

The μ and σ are mean and standard deviation in Hamming distances and derived from a Gaussian fit to the histogram. Embedded PUFs lead to μ of 0.5, σ of 0.032, and DoF value of 227. The encoding capacity is calculated as 2^{227} = 226810.

Calculation of the impact pressure for the water impact test

The impact pressure of the water droplets was calculated using equation (S6) given below for the continuous water impact test.

$$p = \frac{1}{2} \times \rho \times v^2 \tag{S6}$$

p: Impact pressure (Pa) ρ: Density (1000 kg/m^3) v: Velocity of water (m/s)

Calculation of the velocity

- For the water spraying impact and continuous water stream impact tests, the velocity of water was calculated from the measured volumetric flow rate using equation (S7).

$$\mathrm{v} = \frac{Q}{A} \tag{S7}$$

Q: Volumetric flow rate (m^3/s) A: Cross-sectional area of the nozzle (m^2)

Q = 1.2×10^{-4} m^3/s for the continuous water stream impact

A = $\pi\times (2.89\times10^{-3}\ \mathrm{m})^2 = 2.62\times10^{-5}\ \mathrm{m}^2$ for the continuous water stream impact

Table S2. Impact velocity and pressure for the continuous water impact test

Impact Test	Impact velocity (m/s)	Impact Pressure (Pa)
Continuous water stream	4.58	10488.0

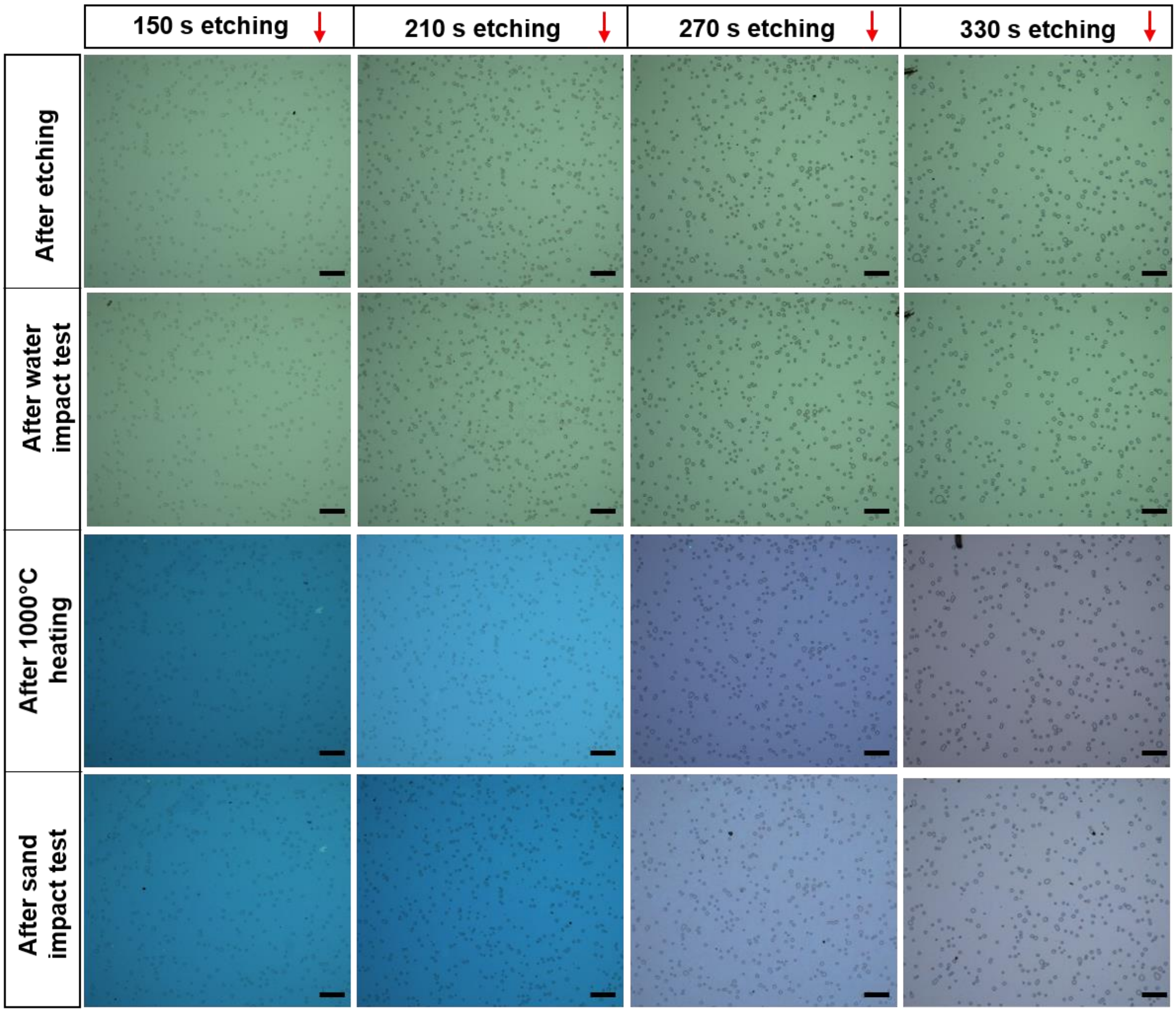

Figure S4. Optical microscope images of surface embedded PUFs before and after the tests. Scale bars: 100 µm.

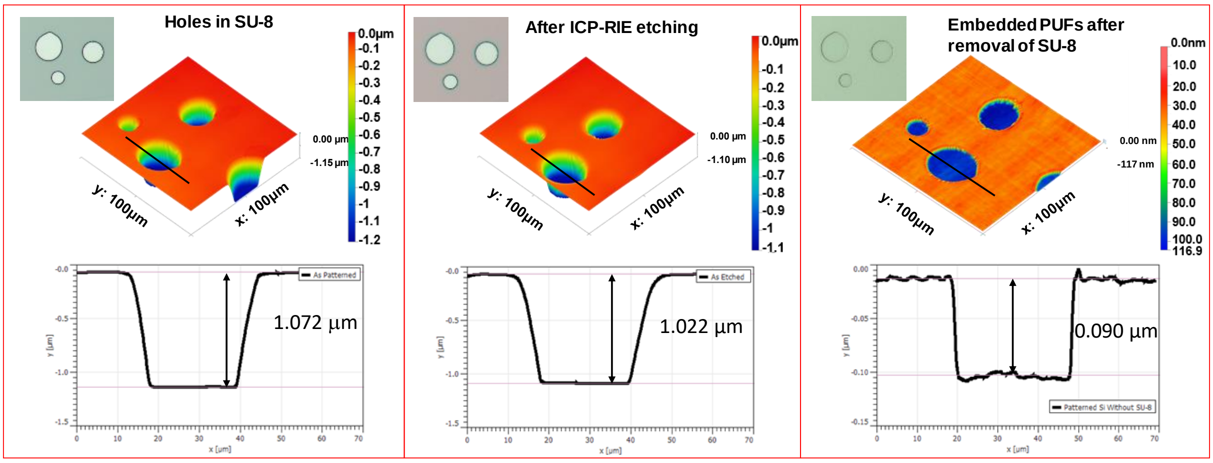

Figure S5. AFM images taken on the exact same position after opening of holes in SU-8 film, dry etching and burning of SU-8. Height profiles were measured to determine the etching rate for SU-8 and silicon under identical conditions.
